# The effect of an Internet-based digital storytelling intervention on depression literacy, stigma, and help-seeking attitudes among university students: A randomized controlled trial

**DOI:** 10.1016/j.invent.2026.100910

**Published:** 2026-02-20

**Authors:** Ömer Özer

**Affiliations:** Anadolu University, Guidance and Psychological Counseling Department, Eskisehir, Turkiye

**Keywords:** Digital storytelling, Depression literacy, College students

## Abstract

This randomized controlled trial evaluated the effectiveness of an internet-based digital storytelling intervention (DST) on depression literacy, stigma, and help-seeking attitudes among first-year university students. A total of 261 students (63.6% female, Mage = 19.8) were randomly assigned to the DST intervention (*n* = 119) or a control group (*n* = 142). The intervention consisted of five short narrative-based videos portraying lived experiences with depression, viewed over a four-week period. Self-report measures of depression literacy, stigma, social stigma regarding help-seeking, and attitudes toward seeking psychological help were collected at baseline, post-test, and three-month follow-up. Compared to the control group, the intervention group demonstrated significant improvements in depression literacy (η^2^_p_ = 0.03) and help-seeking attitudes (η^2^_p_ = 0.04), with effects sustained at follow-up. No significant effects were found for reductions in general stigma or perceived social stigma. Exploratory analyses indicated that increases in depression literacy were associated with more positive attitudes toward professional help. Findings suggest that internet-based digital storytelling can effectively enhance depression literacy and promote help-seeking intentions among first-year students, although its impact on stigma appears limited. Future research should investigate tailoring interventions with demographically relevant narratives and stigma-focused content to maximize effectiveness.

## Highlights

• The study evaluates a 4-week, internet-based digital storytelling intervention targeting university students.
• The intervention led to significant increases in depression literacy.
• The intervention improved attitudes toward seeking professional psychological help.
• The intervention did not significantly reduce general stigma or social stigma related to help-seeking.
• Effects on literacy and help-seeking attitudes were sustained at 3-month follow-up.

## 1 Introduction

University years are a time filled with excitement and new experiences. However, they also present significant challenges as students undergo developmental, academic, social, emotional, and lifestyle changes during this stage of life (Worsley et al., 2021). Research consistently indicates that mental health difficulties are relatively common among university students (Pedrelli et al., 2015). While some scholars have described these challenges as a developmental “crisis,” this terminology is often used to underscore their importance rather than to imply an acute or exceptional state (Patel and Lewis, 2023; Siegel et al., 2023). Emerging adulthood is widely recognized as a period during which mental disorders frequently first appear (Arnett et al., 2014; McGorry et al., 2024), and many students experience conditions such as depression, anxiety, or substance use problems during this time (Bruffaerts et al., 2018; Solmi et al., 2022).

Large-scale, multinational studies conducted by the World Health Organization similarly report that approximately one-third of first-year university students meet diagnostic criteria for at least one mental disorder, although only a minority access professional support (Auerbach et al., 2018; McCabe et al., 2024; Seyfi et al., 2013). Depression, in particular, has consistently emerged as a prominent concern. Although one meta-analysis estimated the prevalence of depression among university students at 25%, Sheldon et al. (2021) caution that comparisons with general population estimates—such as the often-cited 12.9%—should be interpreted carefully due to methodological variation across studies. Even so, accumulating evidence suggests that mental health difficulties during the university years can negatively affect academic performance and increase dropout risk (Bruffaerts et al., 2018; Sinval et al., 2024). Accordingly, promoting student mental well-being and strengthening support mechanisms for those at risk remain important priorities across higher education settings worldwide.

Despite the high prevalence of mental health problems among university students, help-seeking behavior remains relatively low. Stigma—both perceived (social) and internalized (personal)—represents one of the most prominent barriers to seeking professional help (Eisenberg et al., 2009; Golberstein et al., 2008; Martin, 2010). Personal stigma, in particular, significantly reduces both the willingness to seek help and actual help-seeking behaviors. Systematic reviews consistently identify stigma as one of the most influential attitudinal barriers, especially for young people, men, and ethnic minorities (Clement et al., 2015). Beyond stigma, self-concealment and a lack of trust in professional services further discourage students from pursuing mental health care (Masuda et al., 2012). One of the most effective strategies to mitigate such barriers is increasing knowledge and awareness through Mental Health Literacy (MHL). Initially defined as “knowledge and beliefs about mental disorders which aid their recognition, management, or prevention” (Jorm et al., 1997), MHL encompasses the cognitive and social skills enabling individuals to access, understand, and apply accurate information (Kutcher et al., 2016). It promotes help-seeking by helping individuals recognize their issues, learn about evidence-based interventions, and develop appropriate attitudes for seeking support (Jorm, 2012; Jorm et al., 1997; Özel and Duzcu, 2018). Importantly, MHL addresses stigma while empowering individuals to protect their mental well-being (Kutcher et al., 2016). This is particularly vital for university students, as accurate information during this high-risk period helps reduce stigma and encourages help-seeking actions.

Depression represents a critical domain for the application of MHL among university students (Reichel et al., 2021). Enhancing depression literacy increases awareness, fosters positive help-seeking attitudes, and reduces stigma (Al-Shannaq et al., 2023; Conceição et al., 2022). Without adequate MHL, individuals may normalize symptoms, delay help-seeking, or conceal struggles—especially when stigma is internalized—potentially worsening their condition (Jorm, 2000; Jorm et al., 2012). At the individual level, increased MHL enhances well-being and quality of life (Elkin et al., 2025); societally, it fosters community health and decreases healthcare costs through early intervention (Jorm et al., 2012; Kutcher et al., 2016).

### 1.1 Digital storytelling to overcome stigma and foster help-seeking attitudes

To effectively deliver MHL interventions, technology-based approaches have demonstrated promising outcomes (Mohr et al., 2013), with notable increases in adoption during the COVID-19 pandemic (Borghouts et al., 2021). Digital interventions can effectively enhance MHL, improve knowledge and help-seeking behaviors, strengthen self-care capabilities (Brijnath et al., 2016; Nazari et al., 2023; Tay et al., 2020; Tian et al., 2024), and reduce stigma (Amado-Rodríguez et al., 2022; Sun et al., 2025). Their advantages include accessibility, cost-effectiveness, and anonymity compared to traditional face-to-face methods (Andersson and Titov, 2014). Within this digital landscape, digital storytelling—short audiovisual narratives sharing personal experiences—has emerged as a distinctive tool.

Video-based interventions effectively reduce mental health stigma among young people, potentially more than traditional educational methods (Kosyluk et al., 2021). Personal narratives foster empathy, challenge stereotypes, and convey hopeful recovery messages (Keum and Ogrodniczuk, 2023). This effectiveness may stem from contact theory: sharing lived experiences of mental health issues and recovery reduces intergroup prejudice and promotes positive attitudes (Corrigan et al., 2013; Corrigan and O’Shaughnessy, 2007; Song et al., 2011). Empirical studies demonstrate that psychoeducational videos enhance depression literacy and help-seeking intentions among university students (Conceição et al., 2022), while brief personal narrative videos reduce stigma and increase treatment willingness among adolescents and young adults (Amsalem and Martin, 2022). For instance, a pilot study showed that short videos significantly improved symptom recognition and treatment knowledge (Durán et al., 2021). Although some research found no additional benefit over control conditions (Chow et al., 2024), the broader literature underscores digital storytelling's potential as a scalable approach. Given that depression is widespread during university years and stigma hinders help-seeking, this study aims to examine the impact of a digital storytelling intervention on university students' depression literacy, help-seeking attitudes, and stigma levels.

### 1.2 Current study

Building on previous research highlighting the high prevalence of depression among university students and the significant role of stigma in hindering help-seeking underscores the urgent need for innovative interventions in this area. Therefore, the present study aims to examine the effectiveness of a digital storytelling intervention in enhancing MHL regarding depression and promoting positive help-seeking attitudes. Although existing studies highlight the potential of digital content to enhance depression literacy, the specific impact of conveying personal experiences through narrative formats has been examined only to a limited extent; thus, employing digital stories can enhance depression literacy, reduce stigma, and foster more positive attitudes toward seeking professional help. This approach has the potential to make a distinctive contribution to literature.

In line with the existing literature, the following hypotheses were formulated for testing in the current study.

Hypothesis 1 Compared to the control group, participants in the intervention group will report significantly higher levels of depression literacy and more positive attitudes toward seeking professional help at post-test and follow-up.

Hypothesis 2 Compared to the control group, participants in the intervention group will report significantly lower levels of general stigma toward mental health issues and perceived social stigma for seeking help at post-test and follow-up.

## 2 Method

### 2.1 Participants

**Fig. 1:**
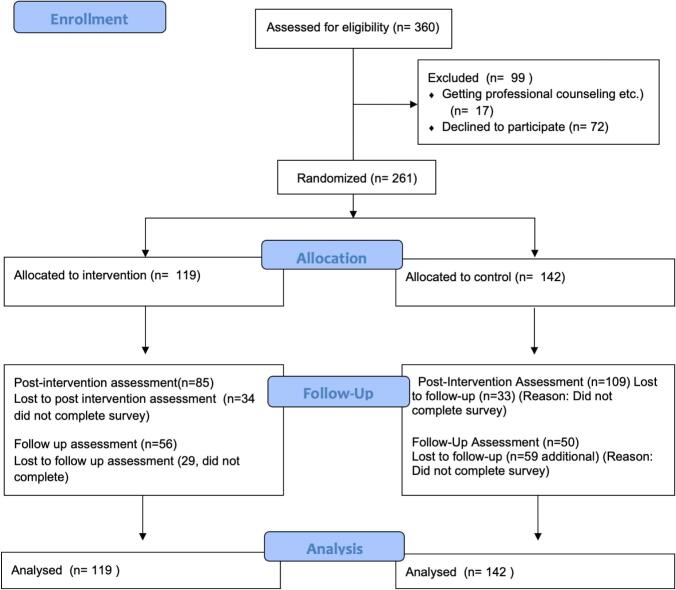
Flow chart.

The participants were first-year students enrolled in various departments of the Faculty of Economics and Administrative Sciences at a middle size public university in Turkiye. All students were enrolled in a typical introductory course, and two classes (each with approximately 180 students) were offered. One class was randomly assigned to the intervention group and the other to the control group. Students who volunteered to participate completed a baseline questionnaire, and those meeting the inclusion criteria were enrolled in the study. Inclusion criteria were: (a) being 18 years or older, (b) not currently receiving professional mental health services, and (c) having access to an internet-enabled device to use online intervention. Written informed consent was obtained from all participants, and the study was approved by the institutional ethics committee (approval date and number masked). A total of 261 students participated in the study, comprising an intervention group (*n* = 119) and a control group (*n* = 142). Participants' ages ranged from 17 to 27 years (M = 19.82). Regarding gender, 95 students (36.4%) were male, and 166 students (63.6%) were female. The number of invited participants, as well as their assignment to groups and their numbers during the pre-intervention, post-intervention, and follow-up stages, are detailed in the Study Flow Diagram (Fig. 1).

### 2.2 Measures

*The Depression Literacy Questionnaire* (*DLQ*) was developed by Griffiths et al. (2004) to measure individuals' knowledge and understanding of depression. The instrument was adapted into Turkish by Göktaş et al. (2022), who also examined its validity and reliability. During the Turkish adaptation, two items (original items 17 and 19) were excluded as they did not adequately fit the factor structure identified in the confirmatory factor analysis. The questionnaire comprises 20 items, each rated on a three-option format (true, false, or do not know). Responses are scored dichotomously, with higher total scores reflecting greater literacy regarding depression. Possible scores range from 0 to 20. The Turkish version demonstrated acceptable internal consistency, with a Cronbach's alpha of 0.71.

The *Attitudes Toward Seeking Psychological Help Scale – Short Form* (ATSPHS-SF) was developed by Fischer and Farina (1995) as an abbreviated version of the original *Attitudes Toward Seeking Professional Psychological Help Scale* (Fischer and Turner, 1970). The short form consists of 10 items rated on a four-point Likert scale ranging from 0 (*disagree*) to 3 (*agree*). Higher scores indicate more favorable attitudes toward seeking professional psychological help. Possible scores range from 0 to 30. The Turkish adaptation of the ATSPHS-SF was conducted by Topkaya (2011), who reported satisfactory psychometric properties. Cronbach's alpha was found to be 0.76, and McDonald's omega was also 0.76, providing evidence for the internal consistency and structural reliability of the scale.

The *Social Stigma for Receiving Psychological Help Scale* (SSRPH) was developed by Komiya et al. (2000) to assess individuals' perceptions of social stigma associated with seeking psychological help. Unlike general stigma toward mental illness, this scale specifically focuses on the stigma attached to the act of seeking professional treatment (e.g. Going to a psychologist due to emotional or interpersonal problems is a sign of personal weakness or inadequacy). The instrument was adapted into Turkish by Topkaya (2011). It consists of five items rated on a four-point Likert scale (1 = *strongly disagree* to 4 = *strongly agree*). Total scores range from 5 to 20, with higher scores indicating stronger perceptions that one would be stigmatized by society when seeking help from a mental health professional. In contrast, lower scores indicate weaker perceptions of such stigma. The Turkish adaptation reported a Cronbach's alpha coefficient of 0.80, demonstrating good internal consistency.

The *Stigma Scale* was developed by Yaman and Güngör (2013) to assess individuals' levels of stigma toward mental health issues. The instrument consists of 22 items organized into four dimensions—*discrimination and exclusion*, *labeling*, *prejudice*, and *psychological health*. This scale measures generalized negative attitudes, prejudice, and social distance toward individuals with mental health problems, rather than the help-seeking process itself (e.g. I don't want to communicate with someone whose sexual preferences I believe to be different.) Items are rated on a five-point Likert scale ranging from 1 (*strongly disagree*) to 5 (*strongly agree*). Possible scores range from 22 to 110, with higher scores reflecting greater levels of stigma. A total score below 55 indicates a low level of stigma, while scores above this threshold suggest higher levels of stigma. The scale demonstrated good internal consistency, with a Cronbach's alpha of 0.84.

### 2.3 Intervention

The intervention consisted of five narrative-based digital stories designed to enhance mental health literacy and attitudes toward seeking help for depression. The digital stories were initially developed by professionals in psychiatry, psychology, and counseling for a separate project. Draft scripts were written by experienced clinicians drawing on their expertise in depression and its treatment. To ensure accuracy, clarity, and appropriateness for the target audience, six subject-matter experts reviewed the scripts, and revisions were made based on their feedback before finalization. The narratives were then performed by professional actors, filmed under the supervision of a director, and carefully edited to produce high-quality, engaging materials for use in this study. All stories were written by mental health professionals based on commonly observed depression presentations. They were not created by individuals with lived experience of depression, which should be considered when interpreting the contact-related mechanisms of the intervention.

After creating an account on a secure online platform with their email address and a password, participants provided demographic information (age and gender) and completed the baseline measures. Students were given four weeks to view all videos. To prevent binge viewing, the platform was designed to limit access to only one video per day, with a minimum interval of two days required between videos. Each story combined a personal account of depression with concise psychoeducational content regarding symptoms, risk factors, and evidence-based treatment:

Story 1 – Fatma (47 years, woman): Depicted the onset of depression following caregiving responsibilities, initial reluctance to seek professional support, and gradual recovery through professional support.

Story 2 – Murat (24 years, man): Illustrated a young graduate's severe depressive episode associated with academic pressure and suicidal ideation, highlighting the role of family support, psychotherapy, and pharmacological treatment in recovery.

Story 3 – Ahmet (63 years, man): Presented recurrent, seasonal depressive episodes in a retired teacher, maladaptive coping through alcohol use, and subsequent improvement with psychiatric intervention and antidepressants.

Story 4 – Begüm (23 years, woman): Focused on a university student's depressive symptoms following a relationship breakup, emotional eating, and hopelessness, along with recovery after an overdose through psychotherapy and medication.

Story 5 – Zehra (54 years, woman): Described a long-standing course of depression, including hospitalization and electroconvulsive therapy (ECT), emphasizing the safety and effectiveness of evidence-based treatments.

At the end of each video, participants answered one or two brief open-ended questions. These items were used solely to confirm that the video had been watched and were not intended to assess knowledge or attitudes; therefore, they were excluded from the analyses. After the four-week viewing period, all participants, regardless of completion status, were invited to complete the post-test via the platform. A three-month follow-up assessment was subsequently administered. All data was collected online. Students were informed that they could complete the measures in class if they preferred, but no additional reminders were sent.

Participants in the control group did not receive the intervention during the study period but retained full access to standard university counseling services. Additionally, following the completion of the study, the digital storytelling platform was made accessible to all university students—including the control group—as a permanent preventive mental health resource.

### 2.4 Statistical analysis

All statistical analyses were conducted using R (RStudio 2024.12.1 Build 563; Posit Software, PBC). Linear mixed-effects models were fitted using the lme4 package, and Type III ANOVA tables were obtained via Satterthwaite's method using the lmerTest package. Post hoc pairwise comparisons and estimated marginal means were computed with the emmeans package. Effect sizes (partial eta squared) were calculated using the effect size package. All visualizations were created using the ggplot2 package.

Descriptive statistics (including mean, standard deviation, skewness, and kurtosis) were computed for all outcome variables across measurement points using the psych and moments packages in R. The skewness and kurtosis values were examined to evaluate the normality of the distributions. All values fell within acceptable limits for parametric analysis (i.e., skewness < |1|, kurtosis < |2|).

## 3 Results

### 3.1 Initial analyses

Descriptive statistics were calculated for all outcome variables, including stigma, depression literacy, social stigma related to psychological help-seeking, and attitudes toward seeking help, across pre-test, post-test, and follow-up assessments. The statistics reported include the number of valid observations (n), minimum and maximum values, means, standard deviations, skewness, and kurtosis. The results are presented in Table 1.

**Table 1:** Descriptive statistics for stigma, depression literacy, social stigma regarding help-seeking, and attitudes toward seeking help in the groups across pre-test, post-test, and follow-up.

Variable	n	Min	Max	Mean	Sd	Skewness	Kurtosis
Intervention Group							
Stigma – Pre Test	119	26	101	49.34	11.75	0.950	2.760
Stigma – Post Test	85	23	73	45.94	10.47	0.344	−0.292
Stigma – Follow Up	56	28	76	44.89	11.64	0.557	−0.197
Depression Literacy – Pre Test	119	7	20	13.99	2.76	−0.424	−0.086
Depression Literacy – Post Test	85	7	20	15.33	2.77	−0.432	0.290
Depression Literacy – Follow Up	56	7	20	15.57	3.48	−0.702	0.071
Social Stigma Regarding Help-Seeking – Pre Test	119	5	16	9.91	2.81	0.053	−0.669
Social Stigma Regarding Help-Seeking – Post Test	85	5	16	9.22	2.88	0.083	−0.600
Social Stigma Regarding Help-Seeking – Follow Up	56	5	17	9.75	3.29	0.353	−0.908
Attitudes Toward Seeking Help – Pre Test	119	4	29	18.66	5.17	0.089	−0.419
Attitudes Toward Seeking Help – Post Test	85	12	30	20.85	4.21	−0.035	−0.687
Attitudes Toward Seeking Help – Follow Up	56	10	30	20.98	5.03	−0.125	−0.807

Control Group							
Stigma – Pre Test	142	29	88	50.50	9.93	0.605	0.477
Stigma – Post Test	109	29	80	49.65	9.75	0.387	0.031
Stigma – Follow Up	50	25	91	50.38	14.95	0.433	−0.452
Depression Literacy – Pre Test	142	8	19	14.26	2.31	−0.360	−0.167
Depression Literacy – Post Test	109	7	19	14.37	2.47	−0.530	−0.028
Depression Literacy – Follow Up	50	8	19	14.12	2.72	−0.385	−0.610
Social Stigma Regarding Help-Seeking – Pre Test	142	5	16	9.88	2.91	0.080	−0.684
Social Stigma Regarding Help-Seeking – Post Test	109	5	17	9.96	2.93	−0.179	−0.782
Social Stigma Regarding Help-Seeking – Follow Up	50	5	16	10.04	3.12	−0.167	−1.093
Attitudes Toward Seeking Help – Pre Test	142	5	30	19.43	4.80	−0.116	−0.227
Attitudes Toward Seeking Help – Post Test	109	1	30	19.50	5.16	−0.454	0.826

Descriptive statistics for all outcome variables were calculated separately for the intervention and control groups across pretest, post-test, and follow-up measurements (see Table 1). Skewness values ranged from |0.05| to |0.95|, and kurtosis values ranged from |0.08| to |2.76|, indicating approximately normal distributions suitable for parametric analyses.

### 3.2 Stigma

**Fig. 2:**
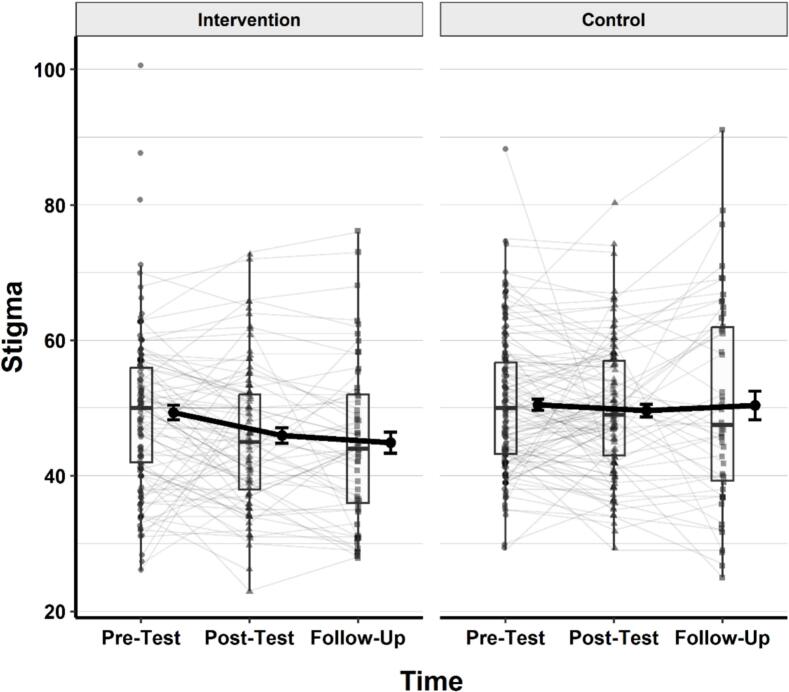
Changes in Stigma Scores Across Time in the Intervention and Control Groups Note. Thin grey lines represent individual participants’ trajectories. Black dots show group means with standard error bars. Thick black lines connect group means over time. Boxplots represent the distribution of scores at each time point, including medians and interquartile ranges. Lower scores reflect lower levels of stigma.

A linear mixed-effects model was conducted to examine the effects of group (intervention vs. control), time (pretest, post-test, follow-up), and their interaction on stigma scores. Participant ID was included as a random intercept to account for within-subject correlation. Type III ANOVA using Satterthwaite's method revealed a significant main effect of group, *F* (1, 276.64) = 3.91, *p* = .049, η^2^_p_ = 0.01, the main effect of time was not significant, *F* (2, 320.25) = 2.92, *p* = .055, η^2^_p_ = 0.02. The interaction between group and time was not statistically significant, *F* (2, 320.25) = 1.92, *p* = .149, η^2^_p_ = 0.01. A visual representation of the changes in stigma (DAM) scores across time and groups is provided in Fig. 2.

Post-hoc comparisons of estimated marginal means (EMMs) were conducted using Holm-adjusted pairwise contrasts. The intervention group had significantly lower stigma scores (*M* = 47.7, 95% CI [45.8, 49.5]) compared to the control group (*M* = 50.2, 95% CI [48.5, 52.0]), *t*(281) = −1.98, *p* = .049.

No statistically significant differences were observed between time points: pretest (*M* = 49.9, 95% CI [48.6, 51.3]) vs. post-test (*M* = 48.4, 95% CI [46.9, 49.9]), *t*(324) = 2.24, *p* = .077; pretest vs. follow-up (*M* = 48.5, 95% CI [46.7, 50.3]), *t*(333) = 1.68, *p* = .188; or post-test vs. follow-up, *t*(316) = −0.10, *p* = .924.

### 3.3 Depression literacy

**Fig. 3:**
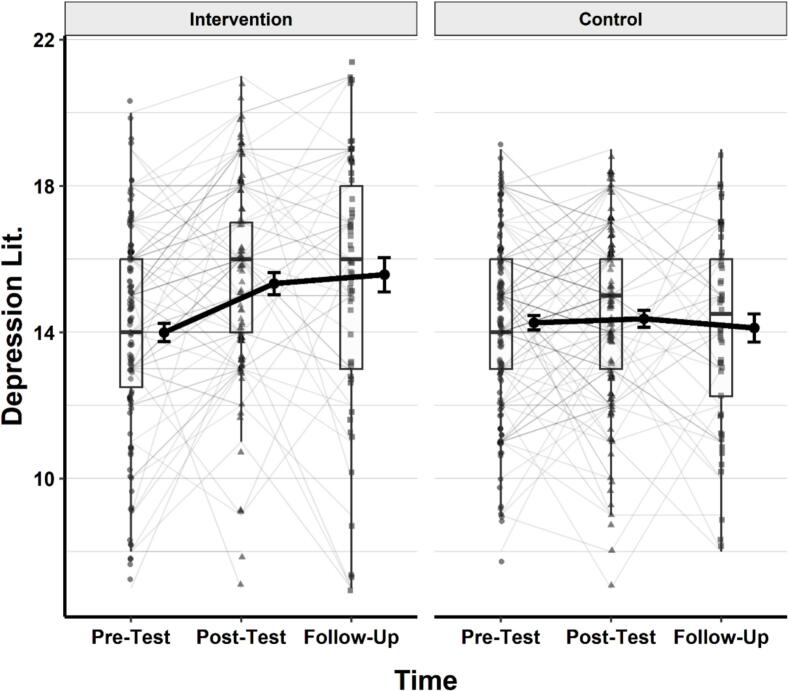
Changes in depression literacy scores across time in the intervention and control groups. *Note.* Thin grey lines represent individual participants' trajectories. Black dots show group means with standard error bars. Thick black lines connect group means over time. Boxplots represent the distribution of scores at each time point, including medians and interquartile ranges. Lower scores reflect lower levels of stigma.

As with stigma, a linear mixed-effects model was conducted to examine the effects of group (intervention vs. control), time (pretest, posttest, follow-up), and their interaction on depression literacy scores. Participant ID was included as a random intercept to account for within-subject correlation. Type III ANOVA using Satterthwaite's method revealed a significant main effect of time, *F* (2, 346.95) = 5.99, *p* = .003, η^2^_p_ = 0.03, and a significant group × time interaction, *F* (2, 346.95) = 4.65, *p* = .010, η^2^_p_ = 0.03. The main effect of group was not statistically significant, *F* (1, 288.30) = 1.97, *p* = .161, η^2^_p_ = 0.007. A visual representation of the changes in depression literacy scores across time and groups is provided in Fig. 3.

Post-hoc comparisons of estimated marginal means (EMMs) were conducted using Holm-adjusted pairwise contrasts. There was no significant overall difference in depression literacy between the intervention group (M = 14.7, 95% CI [14.3, 15.1]) and the control group (*M* = 14.3, 95% CI [13.9, 14.7]), *t* (281) = 1.40, *p* = .161.

Within-group comparisons revealed that the intervention group showed significant gains in depression literacy from pretest to post-test (*t* (340) = −4.09, *p* < .001) and from pretest to follow-up (*t* (353) = −3.12, *p* = .004), with no significant change between post-test and follow-up (*p* = .717). In contrast, no significant differences were observed across time points within the control group (ps = 1.00 for all).

### 3.4 Social stigma regarding help-seeking

**Fig. 4:**
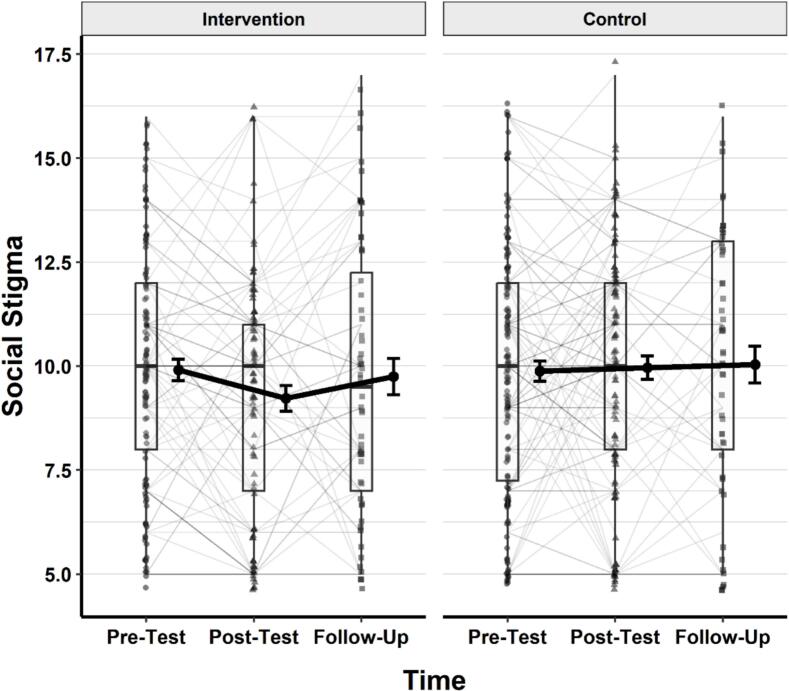
Changes in social stigma related to psychological help-seeking across time in the intervention and control groups. *Note.* Thin grey lines represent individual participants' trajectories. Black dots show group means with standard error bars. Thick black lines connect group means over time. Boxplots represent the distribution of scores at each time point, including medians and interquartile ranges. Lower scores reflect lower levels of stigma.

Continuing with the social stigma related to psychological help-seeking, a linear mixed-effects model was conducted to examine the effects of group, time, and their interaction. Participant ID was included as a random intercept. Type III ANOVA using Satterthwaite's method revealed no significant main effects of group, *F* (1, 285.46) = 1.19, *p* = .277, η^2^_p_ = 0.004, or time, *F* (2, 334.77) = 1.19, *p* = .305, η^2^_p_ = 0.007. The group × time interaction was also not significant, *F* (2, 334.77) = 1.63, *p* = .197, η^2^_p_ = 0.010. A visual representation of the changes in the scores across time and groups is provided in Fig. 4.

### 3.5 Attitudes toward seeking help

**Fig. 5:**
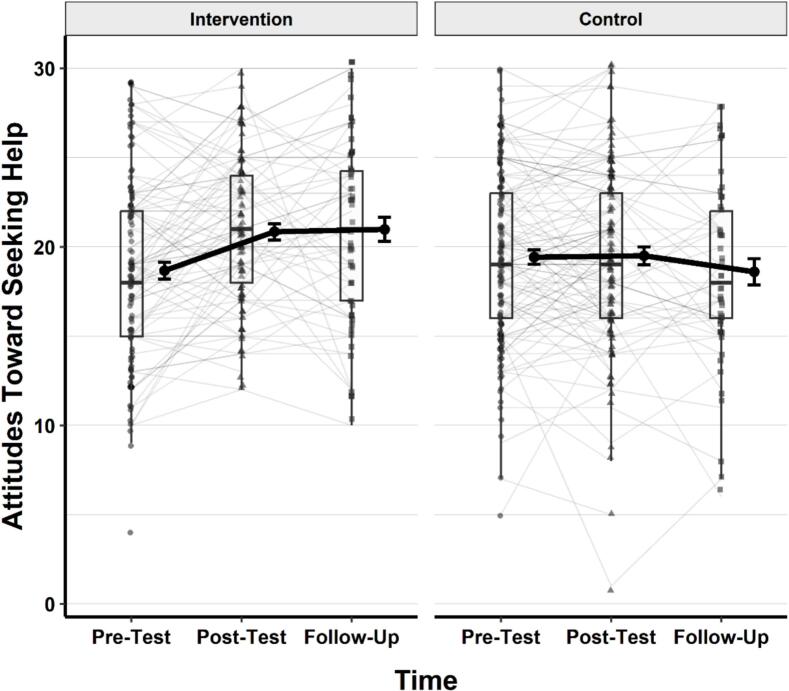
Changes in attitudes toward seeking help across time in the intervention and control groups. *Note.* Thin grey lines represent individual participants' trajectories. Black dots show group means with standard error bars. Thick black lines connect group means over time. Boxplots represent the distribution of scores at each time point, including medians and interquartile ranges. Lower scores reflect lower levels of stigma.

Finally, a linear mixed effects model was conducted to examine the effects of group, time, and their interaction on attitudes toward seeking help. Participant ID was included as a random intercept. Type III ANOVA using Satterthwaite's method revealed a significant main effect of time, *F* (2, 316.44) = 4.28, *p* = .015, η^2^_p_ = 0.03, and a significant group × time interaction, *F* (2, 316.44) = 6.66, *p* = .001, η^2^_p_ = 0.04. The main effect of group was not significant, *F* (1, 275.30) = 0.79, *p* = .374, η^2^_p_ = 0.003. A visual representation of the changes in help-seeking attitudes across time and groups is provided in Fig. 5.

Post-hoc comparisons of estimated marginal means (EMMs) were conducted using Holm-adjusted pairwise contrasts. There was no significant overall difference in attitudes toward seeking help between the intervention group (M = 19.8, 95% CI [18.9, 20.6]) and the control group (M = 19.2, 95% CI [18.4, 20.0]), *t* (280) = 0.89, *p* = .374.

Within-group comparisons showed that the intervention group demonstrated significant increases in attitudes toward seeking help from pretest (M = 18.7) to post-test (M = 20.4), *t* (323) = −4.01, *p* < .001, and from pretest to follow-up (M = 20.3), *t* (328) = −3.23, *p* = .003. No significant difference was observed between post-test and follow-up (*p* = .856).

In contrast, the control group showed no significant differences in scores across time (*all ps* = 1.00).

## 4 Discussion

The present study evaluated the effectiveness of a digital storytelling intervention designed to enhance MHL regarding depression, promote positive help-seeking attitudes, and reduce stigma among university students. The findings offer mixed support for the efficacy of the intervention. In line with the study's hypotheses, the intervention led to significant improvements in participants' depression literacy and attitudes toward seeking professional help. However, contrary to expectations, it failed to produce a significant reduction in either general stigma toward mental health issues or the perceived social stigma associated with seeking help.

The analysis revealed that participants in the intervention group demonstrated a significant increase in depression literacy from pre-test to post-test, with these gains being maintained at the three-month follow-up. In contrast, the literacy levels for depression in the control group remained unchanged throughout the study. This finding aligns with a pilot study conducted with university students, which also reported an increase in participants' ability to recognize depression symptoms and identify helpful treatment strategies following a video-based intervention (Durán et al., 2021). Furthermore, the results are consistent with a broader body of literature indicating the effectiveness of video interventions on various dimensions of MHL (Ito-Jaeger et al., 2022; Keum et al., 2022; Niederkrotenthaler et al., 2022). This suggests that the established efficacy of digital storytelling in general learning contexts (Kim and Li, 2021; Robin, 2008) also extends to conveying mental health information, positioning it as a valuable tool for both patient education and preventive interventions. Hinyard and Kreuter (2007) also noted that narrative communication—defined as a coherent story with characters, conflict, and resolution—can serve as an important tool in psychological help because, unlike traditional health messaging based on statistics and logic, it enables people to receive information more comfortably and familiarly.

Another significant impact of the intervention was the positive change in participants' attitudes toward seeking professional help, an effect that was maintained at the three-month follow-up. This result provides strong support for the second part of the study's first hypothesis. This finding is consistent with the results of a randomized controlled trial by Amsalem and Martin (2022), which similarly used brief personal narrative videos with adolescents and found that the intervention significantly increased participants' treatment-seeking intentions. The success of the intervention in this domain can be attributed to the hopeful recovery narratives presented in the stories. As Amsalem and Martin (2022) emphasize, stories of peer characters who seek and benefit from treatment can “humanize” the experience of depression, thereby normalizing and encouraging help-seeking behavior. Similarly, the digital stories in the present study, which featured characters who were initially reluctant to seek professional support but recovered with treatment, likely had a positive influence on participants' attitudes. This outcome also aligns directly with the findings of a study by Kruger et al. (2022), which used “contact videos” targeted at university students and reported that the intervention significantly increased participants' intentions to seek counseling. Kruger et al. (2022) attribute this success to the message being more relatable and impactful when delivered by peers of a similar social status. It is plausible that the digital stories in this study, particularly through characters like “Begüm”—a university student experiencing depressive symptoms after a relationship breakup—leveraged a similar peer modeling mechanism, thereby shaping help-seeking attitudes in a positive direction. Therefore, observing peers discredit negative stereotypes about treatment and help-seeking likely served as a powerful incentive for the viewers.

Furthermore, the positive change in help-seeking attitudes can be interpreted as a natural consequence of the intervention's success in increasing depression literacy. As established in the theoretical framework, MHL promotes help-seeking behavior by enabling individuals to learn about evidence-based interventions (Gorczynski et al., 2017; Lien et al., 2024; Lui et al., 2024; Wei et al., 2013). In this context, it can be argued that the intervention first enhanced participants' knowledge that depression is a treatable condition and that effective treatments are available. As a direct result of this new and accurate information, participants' initial attitudes of uncertainty or negativity toward professional help may have evolved into a more positive and accepting stance. In other words, when individuals learn what to expect and that treatment can be effective, they become more willing to pursue it.

The study's second hypothesis predicted that the digital storytelling intervention would reduce both general stigma toward mental health issues and perceived social stigma related to seeking professional help. However, the analysis results did not support this hypothesis for either type of stigma. The findings showed that the change in stigma scores over time for the intervention group was not significantly different from that of the control group. This indicates that the digital storytelling intervention did not have a measurable effect on the study's stigma-related outcomes. These results are inconsistent with those of Zhuang and Guidry (2022), who found in their meta-analytic study that storytelling had a small but significant effect on reducing stigma. Fong and Mak (2022) also reported nuanced findings regarding the effectiveness of digital storytelling in reducing stigma. Their research found that stigma-related content in internet-based storytelling interventions led to a notable decrease in public stigma; however, the participants' scores concerning their willingness or intention to engage behaviorally with individuals experiencing mental health issues remained unaffected.

Although the current study's findings appear to differ from those of the previously mentioned studies, they also align with other research indicating that psychoeducational or web-based interventions often fail to reduce stigma. For instance, Nazari et al. (2023) systematically examined existing web-based educational interventions aimed at increasing MHL, reducing stigma, and improving help-seeking intentions or attitudes among young people. The studies included in their meta-analysis and systematic review showed that these interventions enhance MHL but do not decrease stigma levels. Researchers have discussed how stigma can resist change in response to such interventions because it is often rooted in deeply held beliefs and values (Link and Phelan, 2001). As widely discussed in the literature, stigma is one of the main barriers to seeking help, especially among young people (Clement et al., 2015; Gulliver et al., 2010; Schnyder et al., 2017); therefore, as an ongoing challenge and emotional and social barrier to seeking psychological help, participants' stigma levels in the current study may be less likely to be influenced by the digital storytelling intervention. Additionally, similar to the points raised by Nazari et al. (2023), the lack of tailoring in the interventions may also contribute to minimal or no significant reduction in stigma, as they might not address the specific needs or preferences of the participants. It is also important to consider the baseline characteristics of the sample to contextualize these findings. The mean baseline stigma score for the intervention group was 49.34, which is already below the scale's cut-off point of 55 indicating low stigma levels. This suggests a potential ‘floor effect,’ where participants entered the study with relatively positive attitudes, leaving limited room for further improvement compared to populations with higher initial stigma levels.

Furthermore, Fong and Mak (2022) found that stigma-related content was a crucial factor in digital storytelling interventions designed to reduce stigma. However, the stories used in the current intervention did not specifically include content focused on stigma. Therefore, the lack of a significant effect of the current intervention on participants' stigma-related outcomes could also be due to its more indirect approach to stigma reduction. Another possible reason for the non-significant impact of digital storytelling on participants' stigma-related outcomes could be related to the demographic characteristics of the individuals featured in the intervention videos. For example, although two of the five storytellers (Begüm and Murat) were roughly the same age as the participants, three of them were around their 50s or older. Prior research has indicated that viewers generally exhibit greater empathy and stronger identification with characters who possess similar demographic characteristics to their own, including age (Cohen, 2001; McKeever, 2015; Moyer-Gusé, 2008). In the present study, the limited portrayal of storytellers' similarity to the participants may have diminished students' capacity to connect or identify with the narrators/narratives, consequently weakening the potential efficacy of the intervention in reducing stigma.

Finally, considering the broader implications, digital storytelling represents a scalable and cost-effective intervention, which is particularly relevant in the context of this study. University psychological counseling and guidance centers in Turkiye often lack a standardized organizational structure and frequently face personnel shortages. In this landscape, the digital storytelling intervention offers a promising solution as a standardized preventive service. It can be widely deployed to reach a large number of students, effectively bridging the gap between the high prevalence of mental health issues and limited institutional resources, without placing additional strain on existing staff. However, scaling up such tools requires addressing structural barriers, such as establishing clear referral pathways to face-to-face care for those identified as high-risk.”

### 4.1 Limitations and future directions

Although this study has several strengths, including its pre-test post-test control group design involving young adults, it also has notable limitations. This trial was not prospectively registered in a public trial registry, which limits transparency and should be considered when interpreting the findings.

Firstly, an a priori power analysis was not conducted to determine the required sample size, which limits the assessment of statistical power. Secondly, the participants in the current study were predominantly female students in their early twenties (first-year students) from a single faculty at a public university; therefore, the findings may only be applicable to those individuals with similar characteristics. Voluntary participation may also introduce self-selection bias, as pre-existing interest in mental health topics could increase individuals' likelihood of participation. Furthermore, comprehensive demographic data were not collected. As this study was designed as a universal preventive intervention targeting the shared developmental transition of all first-year students, the primary aim was to assess general efficacy across the cohort rather than focusing on specific subgroups. Additionally, limiting these questions helped minimize participant burden and barriers to entry for this broad audience. Additionally, access to an internet-enabled device to participate in the study could potentially exclude individuals from lower socioeconomic backgrounds. Consequently, further research would benefit from achieving a more balanced gender distribution and greater diversity among participants in terms of age, academic level, and socioeconomic status, along with employing a larger sample size, which could increase the generalizability of the results. Thirdly this study showed that while the intervention was effective in improving depression literacy and attitudes toward psychological help, the results failed to show the impact of the intervention on general stigma toward mental health issues and perceived social stigma related to seeking professional help, which could result from the lack of tailoring in intervention and having storytellers from diverse ages that could restrict the participants' identification with the narrations. Therefore, future studies are recommended to incorporate more individually tailored interventions, while also utilizing narratives or narrators that reflect participants' demographic characteristics, so that they can facilitate identification and, consequently, have a greater impact on stigma reduction. Furthermore, narratives that more directly address stigma in digital storytelling interventions could have a greater impact on reducing stigma in the mental health field. Fourth, the intervention design restricted access to one video every two days to prevent ‘binge-watching’ and encourage spaced learning. However, this restriction may not reflect how young people typically engage with digital media and could have impacted user engagement. Finally, although adherence was tracked via video completion rates (71.4% of the intervention group completed the program), I did not systematically collect qualitative data on participant satisfaction or user experience, which limits our understanding of the intervention's acceptability. Fifth, this study assessed attitudes toward help-seeking but did not measure actual help-seeking behaviors or specific behavioral intentions to utilize mental health services. While improved attitudes are often a precursor to behavior change, this study cannot confirm whether these changes translated into actual service use.

The last limitation relates to the nature of the digital stories used in the intervention. Although the narratives were written by mental health professionals to ensure clinical accuracy and coherence, they were not created by individuals with lived experience of depression. Lived-experience involvement is often considered important in contact-based stigma reduction approaches, as it may enhance authenticity, emotional resonance, and perceived credibility. Therefore, this absence should be taken into account when interpreting the potential impact of the intervention on stigma outcomes.

Despite some limitations, this study offers valuable insights into how digital storytelling can impact MHL for depression, help-seeking attitudes, and the stigma levels of university students or emerging adults. The results are encouraging, indicating that these interventions have significant potential to increase MHL and promote positive attitudes toward seeking support. Moving forward, future research can not only build on these findings but also expand them by investigating how these interventions yield results with different demographic groups and mental health concerns, thereby broadening and enhancing our understanding to make it even more inclusive. We used the CONSORT reporting guideline (1) to draft this manuscript, and the CONSORT reporting checklist (2) when editing, included in supplement A. The data are available from the corresponding author upon reasonable request.

## Declaration of competing interest

The author declare that they have no known competing financial interests or personal relationships that could have appeared to influence the work reported in this paper.

## References

[bb0010] Al-Shannaq Y., Jaradat D., Ta’an W.F., Jaradat D. (2023). Arch. Psychiatr. Nurs..

[bb0015] Amado-Rodríguez I.D., Casañas R., Mas-Expósito L., Castellví P., Roldan-Merino J.F., Casas I., Lalucat-Jo L., Fernández-San Martín M.I. (2022). Effectiveness of mental health literacy programs in primary and secondary schools: a systematic review with meta-analysis. Children.

[bb0020] Amsalem D., Martin A. (2022). Reducing depression-related stigma and increasing treatment seeking among adolescents: randomized controlled trial of a brief video intervention. J. Child Psychol. Psychiatry.

[bb0025] Andersson G., Titov N. (2014). Advantages and limitations of Internet-based interventions for common mental disorders. World Psychiatry.

[bb0030] Arnett J.J., Žukauskienė R., Sugimura K. (2014). The new life stage of emerging adulthood at ages 18-29 years: implications for mental health. The Lancet.

[bb0035] Auerbach R.P., Mortier P., Bruffaerts R., Alonso J., Benjet C., Cuijpers P., Demyttenaere K., Ebert D.D., Green J.G., Hasking P., Murray E., Nock M.K., Pinder-Amaker S., Sampson N.A., Stein D.J., Vilagut G., Zaslavsky A.M., Kessler R.C., WHO WMH-ICS Collaborators (2018). WHO World Mental Health Surveys International College Student Project: prevalence and distribution of mental disorders. J. Abnorm. Psychol..

[bb0040] Borghouts J., Eikey E., Mark G., Leon C., Schueller S.M., Schneider M., Stadnick N., Zheng K., Mukamel D., Sorkin D.H. (2021). Barriers to and facilitators of user engagement with digital mental health interventions: systematic review. J. Med. Internet Res..

[bb0045] Brijnath B., Protheroe J., Mahtani K.R., Antoniades J. (2016). Do web-based mental health literacy interventions improve the mental health literacy of adult consumers? Results from a systematic review. J. Med. Internet Res..

[bb0050] Bruffaerts R., Mortier P., Kiekens G., Auerbach R.P., Cuijpers P., Demyttenaere K., Green J.G., Nock M.K., Kessler R.C. (2018). Mental health problems in college freshmen: prevalence and academic functioning. J. Affect. Disord..

[bb0055] Chow G.M., Bird M.D., Cox C., Cooper B.T., Gabana N.T. (2024). A brief web-based depression literacy, efficacy, and stigma intervention among college students. Adv. Ment. Health.

[bb0060] Clement S., Schauman O., Graham T., Maggioni F., Evans-Lacko S., Bezborodovs N., Morgan C., Rüsch N., Brown J.S.L., Thornicroft G. (2015). What is the impact of mental health-related stigma on help-seeking? A systematic review of quantitative and qualitative studies. Psychol. Med..

[bb0065] Cohen J. (2001). Defining identification: a theoretical look at the identification of audiences with media characters. Mass Commun. Soc..

[bb0070] Conceição V., Rothes I., Gusmão R. (2022). The effects of a video-based randomized controlled trial intervention on depression stigma and help-seeking attitudes in university students. Psychiatry Res..

[bb3000] Corrigan P.W., Kosyluk K.A., Rüsch N. (2013). Reducing self-stigma by coming out proud. Am. J. Public Health.

[bb0075] Corrigan P.W., O’Shaughnessy J.R. (2007). Changing mental illness stigma as it exists in the real world. Aust. Psychol..

[bb0085] Durán L., Almeida A.M., Figueiredo-Braga M. (2021). Digital audiovisual contents for literacy in depression: a pilot study with university students. Proc. Comput. Sci..

[bb0090] Eisenberg D., Downs M.F., Golberstein E., Zivin K. (2009). Stigma and help seeking for mental health among college students. Med. Care Res. Rev..

[bb0095] Elkin N., Mohammed A.K., Kılınçel Ş., Soydan A.M., Tanrıver S.Ç., Çelik Ş., Ranganathan M. (2025). Mental health literacy and happiness among university students: a social work perspective to promoting well-being. Front. Psych..

[bb0100] Fischer E.H., Farina A. (1995). Attitudes toward seeking professional psychological help: a shortened form and considerations for research. J. Coll. Stud. Dev..

[bb3005] Fischer E.H., Turner J.L. (1970). Orientations to seeking professional help: development and research utility of an attitude scale. J. Consult. Clin. Psychol..

[bb0105] Fong T.H.C., Mak W.W.S. (2022). The effects of Internet-based storytelling programs (amazing adventure against stigma) in reducing mental illness stigma with mediation by interactivity and stigma content: randomized controlled trial. J. Med. Internet Res..

[bb0110] Göktaş S., Yenilmez Ç., Metintaş S. (2022). Evaluation of Turkish validity and reliability of the Depression Literacy Questionnaire (D-LIT-Questionnaire). J. Tepecik Educ. Res. Hospital.

[bb0115] Golberstein E., Eisenberg D., Gollust S.E. (2008). Perceived stigma and mental health care seeking.

[bb0120] Gorczynski P., Sims-schouten W., Hill D., Wilson J.C. (2017). Examining mental health literacy, help seeking behaviours, and mental health outcomes in UK university students. J. Ment. Health Train. Educ. Pract..

[bb0125] Griffiths K.M., Christensen H., Jorm A.F., Evans K., Groves C. (2004). Effect of web-based depression literacy and cognitive–behavioural therapy interventions on stigmatising attitudes to depression: randomised controlled trial. Br. J. Psychiatry.

[bb0130] Gulliver A., Griffiths K.M., Christensen H. (2010). Perceived barriers and facilitators to mental health help-seeking in young people: a systematic review. BMC Psychiatry.

[bb0135] Hinyard L.J., Kreuter M.W. (2007). Using narrative communication as a tool for health behavior change: a conceptual, theoretical, and empirical overview. Health Educ. Behav. Off. Publ. Soc. Publ. Health Educ..

[bb0140] Ito-Jaeger S., Perez Vallejos E., Curran T., Spors V., Long Y., Liguori A., Warwick M., Wilson M., Crawford P. (2022). Digital video interventions and mental health literacy among young people: a scoping review. J. Ment. Health.

[bb3010] Jorm A.F. (2000). Mental health literacy: public knowledge and beliefs about mental disorders. Br. J. Psychol..

[bb0150] Jorm A.F. (2012). Mental health literacy: empowering the community to take action for better mental health. Am. Psychol..

[bb0155] Keum B.T., Ogrodniczuk J.S. (2023). The role of first-person depression storytelling online video on men’s self-stigma of seeking help, traditional masculinity ideology, and psychological help-seeking attitudes. J. Men’s Stud..

[bb3015] Jorm A.F., Korten A.E., Jacomb P.A., Christensen H., Rodgers B., Pollitt P. (1997). "Mental health literacy": a survey of the public’s ability to recognise mental disorders and their beliefs about the effectiveness of treatment. Med. J. Aust..

[bb3020] Jorm A.F., Reavley N.J., Ross A.M. (2012). Belief in the dangerousness of people with mental disorders: a review. Aust. N. Z. J. Psychiatry.

[bb0160] Keum B.T., Hearns M., Agarwal P., Nguyen M. (2022). Online digital storytelling video on promoting men’s intentions to seek counselling for depression: the role of empathy. Int. J. Soc. Psychiatry.

[bb0165] Kim D., Li M. (2021). Digital storytelling: facilitating learning and identity development. J. Comput. Educ..

[bb0170] Komiya N., Good G.E., Sherrod N.B. (2000). Emotional openness as a predictor of college students’ attitudes toward seeking psychological help. J. Couns. Psychol..

[bb0175] Kosyluk K., Marshall J., Conner K., Macias D.R., Macias S., Michelle Beekman B., Her J. (2021). Challenging the stigma of mental illness through creative storytelling: a randomized controlled trial of this is my brave. Community Ment. Health J..

[bb0180] Kruger E., Pitts S.C., Denenny D., DeLuca J.S., Schiffman J. (2022). Efficacy of contact intervention videos on college students’ intentions toward mental health help-seeking. J. Am. Coll. Health.

[bb0185] Kutcher S., Wei Y., Costa S., Gusmão R., Skokauskas N., Sourander A. (2016). Enhancing mental health literacy in young people. Eur. Child Adolesc. Psychiatry.

[bb0190] Lien Y.-J., Chen L., Cai J., Wang Y.-H., Liu Y.-Y. (2024). The power of knowledge: how mental health literacy can overcome barriers to seeking help. Am. J. Orthopsychiatry.

[bb0195] Link B.G., Phelan J.C. (2001). Conceptualizing stigma. Annu. Rev. Sociol..

[bb0200] Lui J.C., Sagar-Ouriaghli I., Brown J.S.L. (2024). Barriers and facilitators to help-seeking for common mental disorders among university students: a systematic review. J. Am. Coll. Health.

[bb0205] Martin J.M. (2010). Stigma and student mental health in higher education. Higher Educ. Res. Develop..

[bb0210] Masuda A., Anderson P.L., Edmonds J. (2012). Help-seeking attitudes, mental health stigma, and self-concealment among African American College students. J. Black Stud..

[bb0215] McCabe M., Byrne M., Gullifer J., Cornish K. (2024). The relationship between university student help-seeking intentions and well-being outcomes. Front. Psych..

[bb3025] McGorry P.D., Mei C., Dalal N., Alvarez-Jimenez M., Blakemore S.-J., Browne V., Dooley B., Hickie I.B., Jones P.B., McDaid D., Mihalopoulos C., Wood S.J., El Azzouzi F.A., Fazio J., Gow E., Hanjabam S., Hayes A., Morris A., Pang E., Paramasivam K., Quagliato Nogueira I., Tan J., Adelsheim S., Broome M.R., Cannon M., Chanen A.M., Chen E.Y.H., Danese A., Davis M., Ford T., Gonsalves P.P., Hamilton M.P., Henderson J., John A., Kay-Lambkin F., Le L.K.-D., Kieling C., Mac Dhonnagáin N., Malla A., Nieman D.H., Rickwood D., Robinson J., Shah J.L., Singh S., Soosay I., Tee K., Twenge J., Valmaggia L., van Amelsvoort T., Verma S., Wilson J., Yung A., Iyer S.N., Killackey E. (2024). The Lancet Psychiatry Commission on youth mental health. Lancet Psychiatry.

[bb0225] McKeever R. (2015). Vicarious experience: experimentally testing the effects of empathy for media characters with severe depression and the intervening role of perceived similarity. Health Commun..

[bb3030] Mohr D.C., Burns M.N., Schueller S.M., Clarke G., Klinkman M. (2013). Behavioral Intervention Technologies: evidence review and recommendations for future research in mental health. Gen. Hosp. Psychiatry.

[bb0230] Moyer-Gusé E. (2008). Toward a theory of entertainment persuasion: explaining the persuasive effects of entertainment-education messages. Commun. Theory.

[bb0235] Nazari A., Garmaroudi G., Foroushani A.R., Hosseinnia M. (2023). The effect of web-based educational interventions on mental health literacy, stigma and help-seeking intentions/attitudes in young people: systematic review and meta-analysis. BMC Psychiatry.

[bb0240] Niederkrotenthaler T., Till B., Kirchner S., Sinyor M., Braun M., Pirkis J., Tran U.S., Voracek M., Arendt F., Ftanou M., Kovacs R., King K., Schlichthorst M., Stack S., Spittal M.J. (2022). Effects of media stories of hope and recovery on suicidal ideation and help-seeking attitudes and intentions: systematic review and meta-analysis. Lancet Public Health.

[bb0245] Özel Y., Duzcu T. (2018). Ruh sağlığı okuryazarlığı. Uluslararası Sosyal Bilimler Dergisi.

[bb0250] Patel B.P., Lewis B. (2023). Responding to the crisis in college mental health: a call to action. J. Pediatr..

[bb0255] Pedrelli P., Nyer M., Yeung A., Zulauf C., Wilens T. (2015). College students: mental health problems and treatment considerations. Acad. Psychiatry.

[bb0260] Reichel J.L., Dietz P., Sauter C., Schneider F., Oenema A. (2021). Is mental health literacy for depression associated with the intention toward preventive actions? A cross-sectional study among university students. J. Am. Coll. Health.

[bb0265] Robin B.R. (2008). Handbook of Research on Teaching Literacy Through the Communicative and Visual Arts, Volume II.

[bb0270] Schnyder N., Panczak R., Groth N., Schultze-Lutter F. (2017). Association between mental health-related stigma and active help-seeking: systematic review and meta-analysis. Brit. J. Psychiatry J. Mental Sci..

[bb0275] Seyfi F., Poudel K.C., Yasuoka J., Otsuka K., Jimba M. (2013). Intention to seek professional psychological help among college students in Turkey: influence of help-seeking attitudes. BMC. Res. Notes.

[bb0280] Sheldon E., Simmonds-Buckley M., Bone C., Mascarenhas T., Chan N., Wincott M., Gleeson H., Sow K., Hind D., Barkham M. (2021). Prevalence and risk factors for mental health problems in university undergraduate students: a systematic review with meta-analysis. J. Affect. Disord..

[bb0285] Siegel K.R., Mobley T.P., Sanderson C.A. (2023). Addressing the college mental health crisis: training students to become effective bystanders. Psychol. Serv..

[bb0290] Sinval J., Oliveira P., Novais F., Almeida C.M., Telles-Correia D. (2024). Exploring the impact of depression, anxiety, stress, academic engagement, and dropout intention on medical students’ academic performance: a prospective study. J. Affect. Disord..

[bb0295] Solmi M., Radua J., Olivola M., Croce E., Soardo L., Pablo G., Il Shin J., Kirkbride J.B., Jones P., Kim J.H., Kim J.Y., Carvalho A.F., Seeman M.V., Correll C.U., Fusar-Poli P. (2022). Age at onset of mental disorders worldwide: large-scale meta-analysis of 192 epidemiological studies. Mol. Psychiatry.

[bb0300] Song D., Lim H., Chung Y.J. (2011). The stigma of mental illness and the way of destigmatization: the effects of interactivity and self-construal. World Academy of Science, Engineering and Technology. International Journal of Social, Behavioral, Educational, Economic, Business and Industrial Engineering.

[bb0305] Sun G., Wang C., Zhang J. (2025). Effectiveness of mental health literacy interventions for adolescents: a systematic review and meta-analysis. SAGE Open.

[bb0310] Tay J.L., Goh S.S., Klainin-Yobas P. (2020). Online HOPE intervention on mental health literacy among youths in Singapore: an RCT protocol. J. Adv. Nurs..

[bb0315] Tian L., Wong E.L., Dong D., Cheung A.W., Cao Y., Mok K.H., Zhou L., Xu R.H. (2024). Improving mental health literacy using web- or app-based interventions: a scoping review. Digit. Health.

[bb0320] Topkaya N. (2011).

[bb0325] Wei Y., Hayden J.A., Kutcher S., Zygmunt A., McGrath P. (2013). The effectiveness of school mental health literacy programs to address knowledge, attitudes and help seeking among youth. Early Interv. Psychiatry.

[bb0330] Worsley J.D., Harrison P., Corcoran R. (2021). Bridging the gap: exploring the unique transition from home, school or college into university. Front. Public Health.

[bb0335] Yaman E., Güngör H. (2013). Damgalama (stigma) ölçeği’nin geliştirilmesi, geçerlilik ve güvenirlik çalışması. Değerler Eğitimi Dergisi.

[bb0340] Zhuang J., Guidry A. (2022). Does storytelling reduce stigma? A meta-analytic view of narrative persuasion on stigma reduction. Basic Appl. Soc. Psychol..

